# Occurrence of Motor Complications and Gait Problems After Introduction of Medical Treatment in Parkinson's Disease

**DOI:** 10.1155/padi/8857969

**Published:** 2025-11-07

**Authors:** Yasushi Osaki, Yukari Morita, Sho Ohtsuru, Tomohiro Shogase, Daiji Yoshimoto, Toshimasa Miyoshi, Tatsuya Ikeda, Yu Hashimoto, Takuya Matsushita

**Affiliations:** Department of Neurology, Kochi Medical School Hospital, Kohasu, Oko-Cho, Nankoku-Shi, Kochi 785-8505, Japan

## Abstract

**Background:**

Although patients with Parkinson's disease eventually experience motor complications and gait problems including falls, introducing the necessity for gait assistance, or freezing of gait, there may be a medication dose at which patients do not experience both in the early stage of the disease.

**Objectives:**

To identify the medication dose at which Parkinson's disease patients, diagnosed and treated at our hospital, did not experience motor complications and gait problems.

**Methods:**

We retrospectively reviewed the clinical course, including motor complications and gait problems, of 119 newly diagnosed patients with Parkinson's disease for 24 months after the introduction of medical treatment. According to the presence of motor complications and/or gait problems, we categorized the patients into Groups 1–3. We estimated the median latency of motor complications or gait problems by Kaplan–Meier survival analysis. We calculated the levodopa equivalent dose.

**Results:**

Group 1 contained 25 patients with neither motor complications nor gait problems; Group 2 contained 40 patients who experienced motor complications first with a median latency of 11 months; and Group 3 contained 54 patients who experienced gait problems first with a median latency of 9 months. There were significant differences in the levodopa equivalent dose at 24 months among the groups: 250 mg in Group 1, 300 mg in Group 2, and 225 mg in Group 3.

**Conclusions:**

Patients with Parkinson's disease receiving a levodopa equivalent dose between 225 and 300 mg did not experience motor complications and gait problems for 24 months after the introduction of medical treatment.

## 1. Introduction

Patients with Parkinson's disease eventually experience motor complications (MC), namely, ON/OFF fluctuations or dyskinesias, after the introduction of medical treatment. Regardless of medical treatment, patients also experience gait problems (GP), including falls, introducing the necessity for gait assistance or freezing of gait. In terms of MC, most cases experience ON/OFF fluctuations earlier than dyskinesias [1]. Lópes et al. reported that 6.3% of cases experience motor fluctuations, whereas 14.1% experience dyskinesias, at 1 year after the introduction of medical treatment [2]. Cilia et al. stated that 20% of cases experience motor fluctuations, whereas 11% experience dyskinesias at the 1-year follow-up [3]. Undertreatment and disease progression cause falls, and patients may need gait assistance. Although rare, patients may experience freezing of gait from the early stage [1]. Lord et al. reported that 79.7% of an incident cohort of Parkinson's disease had fallen over by 54 months and 26.2% reported retrospective falls at baseline [4]. Wilson et al. found that discrete gait impairment continues to progress in Parkinson's disease over 6 years, reflecting a combination of, and potential interaction between, disease-specific progression and age-related changes [5].

From the cognitive impairment aspect, dopaminergic medication can improve cognitive function in some patients, but it may worsen it in others [6]. Furthermore, impaired inhibition can cause freezing of gait and falls in patients with Parkinson's disease [7, 8].

Clearly, patients with Parkinson's disease experience not only MC but also GP during the long disease course, and the optimal medical treatment should aim to provide a long period without experiencing both. Here, we observed the treatment course of patients with Parkinson's disease, newly diagnosed and treated at our hospital, and aimed to identify the medication dose at which they did not experience MC and GP in the early disease course.

## 2. Materials and Methods

### 2.1. Participants

We included 131 patients (63 men and 68 women) with Parkinson's disease. However, we excluded 12 patients who experienced GP either before the first presentation or in the period in which their medication doses were increasing in the very early stage of the treatment course, because we aimed to identify the accurate dose after the titration was finished. Thus, we analyzed 119 patients (60 men and 59 women) with Parkinson's disease who were followed-up at Kochi Medical School Hospital or related hospitals. All patients were diagnosed as Parkinson's disease and started to receive medical treatment between May 2014 and November 2022 and were followed-up regularly for at least 24 months by us. We collected clinical data from all patients during this 24-month period. We diagnosed Parkinson's disease using the UK Parkinson's Disease Brain Bank criteria. We excluded patients if they presented with significant memory impairment (Mini-Mental State Examination score < 24) or dementia with Lewy bodies. The median age at onset was 71 (interquartile range: 64–77) years. Patients with secondary or atypical parkinsonism were excluded.

### 2.2. Ethics Approval

This retrospective study was approved by the Ethics Committee of the Faculty of Medicine, Kochi University (approval no. 2023-52). Informed consent was waived, as the study involved only the retrieval of descriptions and data from medical records.

### 2.3. Clinical Assessments

We reviewed the occurrence of clinical symptoms including falls, necessity for gait assistance, freezing of gait, ON/OFF fluctuations, and dyskinesias in each patient at each visit, and calculated the latencies of each symptom by months. We considered that patients had MC if the Movement Disorder Society-sponsored revision of the Unified Parkinson's Disease Rating Scale part IV 4.1 or 4.3 score was ≥ 1 [9]. Permission from PAR was granted to use the Mini-Mental State Examination scale in this study.

### 2.4. Medication Dose

We reviewed the medication dose of each patient. Prescribed drugs included levodopa, entacapone, opicapone, selegiline (oral), rasagiline, pramipexole (immediate-release), pramipexole (extended-release), ropinirole, ropinirole tape, rotigotine, amantadine hydrochloride (immediate-release), zonisamide, trihexyphenidyl, and istradefylline. To calculate the levodopa equivalent dose (LED), we used the formulas reported by Jost et al. [10], and the formula that 9 mg of rotigotine tape is equivalent to 24 mg of ropinirole tape.

We calculated the LED, levodopa dose (LD), and levodopa intake/day when the patient was introduced to medical treatment (LEDi and LDi). After the introduction of medical treatment, the medication dose was increased in 69 of the 119 patients during their hospital visits; the median duration of this increase was 1 month (range: 1–4 months). Regardless of the duration, we defined the point at which the increase in dose was finished as “stabilized” (stb). We also calculated the LED (LEDstb), LD (LDstb), and levodopa intake/day at stb, when the patient experienced GP (LEDgp, LDgp) or MC (LEDmc, LDmc), and at 6 (LED6m, LD6m), 12 (LED12m, LD12m), 18 (LED18m, LD18m), and 24 (LED24m, LD24m) months after the introduction of medical treatment.

For the patients who had not experienced either GP or MC by 6 months after the introduction of medical treatment, LED6m or LD6m indicates the medication dose at 6 months. For the patients who had experienced either GP or MC before 6 months after the introduction of medical treatment, LED6m or LD6m indicates the medication dose when they experienced either. The same definitions were applied to LED12m or LD12m, LED18m or LD18m, and LED24m or LD24m.

### 2.5. Groups 1–3

The patients were categorized into Groups 1–3 according to the status of GP and MC during the first 24 months after the introduction of medical treatment: Group 1 consisted of patients who had not experienced GP or MC; Group 2 consisted of patients who experienced MC first; and Group 3 consisted of patients who experienced GP first. Supporting Figure 1 shows the clinical course and endpoint, the LED at the endpoint, and summarizes the categorization into Groups 1–3 of nine representative patients.

### 2.6. Statistical Analysis

We performed statistical analyses using IBM SPSS Statistics version 29.0. We analyzed differences in sex and Hoehn–Yahr stage among Groups 1–3 with the chi-square test. We estimated the development of MC or GP and the median disease duration at the development of MC or GP by means of Kaplan–Meier survival analysis. For continuous variables, we used the Kruskal–Wallis test. For categorical variables among the three groups, we used the Kruskal–Wallis test, followed by the Dunn–Bonferroni *post hoc* test. We considered *p* values < 0.05 statistically significant.

## 3. Results

### 3.1. Participant Characteristics

We introduced medical treatment at 10 (interquartile range: 3–20) months after onset. Median LEDi was 135 (100–150) mg, and median LDi was 100 (100–150) mg. One hundred eleven of the 119 patients were started with a levodopa formula. The demographics, including the number of patients with an orthopedic comorbidity, such as a lumbar spine, hip, or knee problem, of Groups 1–3 are summarized in Table 1. There was a significant difference in age at onset among Groups 1–3 (*p* < 0.05). However, there was no significant difference in the other variables listed in Table 1 between Groups 1–3.

### 3.2. Event Survival Curves and Latency to MC or GP

**Figure 1:**
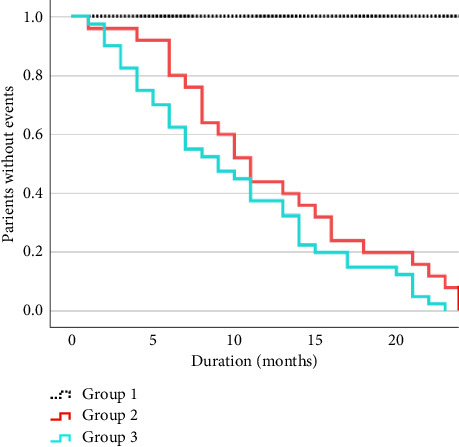
Time-to-event survival curves for Groups 1–3 in relation to disease duration.

Time-to-event survival curves (Figure 1) showed that patients in Group 2 experienced MC at a median latency of 11 months, while patients in Group 3 experienced GP at a median latency of 9 months. No patient in Group 1 experienced either MC or GP until 24 months. In terms of MC, only one patient in Group 2 experienced dyskinesias earlier than ON/OFF fluctuations.

### 3.3. Treatment Course, LED, and Levodopa Intake/Day in Groups 1–3

**Figure 2:**
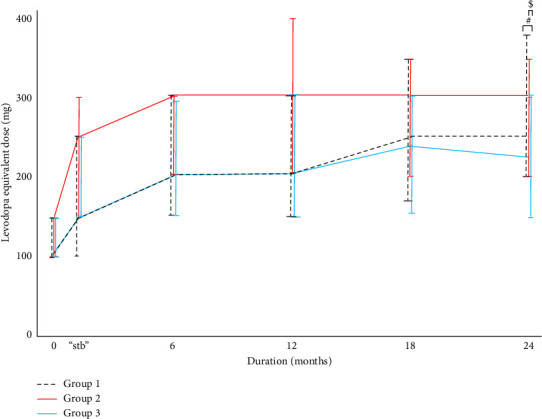
Levodopa equivalent doses in Groups 1–3. Data are median values and vertical bars indicate interquartile range. ^#^*p* < 0.05 between Groups 1–3. ^$^*p* < 0.05 between Groups 2 and 3. stb, the point at which the dosage was stabilized.

The LED in relation to the disease course in Groups 1–3 is shown in Figure 2 and Supporting Table 1. The medication dose was increased in 69 patients after the introduction of medical treatment. From the time of stb to 18 months, there was no significant difference regarding the LED between Groups 1–3. At 24 months, the median LED was 250 mg in Group 1, 300 mg in Group 2, and 225 mg in Group 3; the Dunn–Bonferroni *post hoc* test showed a significant difference in LED24m between Groups 2 and 3 (*p* < 0.05).

The LD in relation to the disease course in Groups 1–3 is shown in Supporting Table 2. There was no significant difference in the LD from the time of stb to 24 months after the introduction of medical treatment.

Table 2 shows levodopa intake/day in relation to disease course in Groups 1–3. There was no significant difference regarding levodopa intake/day between Groups 1–3 from the time of stb to 24 months after the introduction of medical treatment.

## 4. Discussion

We categorized our patients with Parkinson's disease into three groups: Group 1, those who had not experienced GP or MC; Group 2, those who experienced MC first; and Group 3, those who experienced GP first, during the first 24 months after the introduction of medical treatment. There was a significant difference in the LED between Groups 1–3 at 24 months. Regarding the LED, the median LED in Group 1 was increased after 12 months and reached 250 mg at 24 months.

Compared to previous studies [2, 3, 5, 11, 12], the patients in this study were older. Within this study, age at onset was significantly higher in Group 3. In terms of Hoehn–Yahr stage, all patients were stage I or II. There was no significant difference regarding Hoehn–Yahr stage I or II between Groups 1–3.

In Group 3, the LED24m was 225 mg, and the median latency to experience GP was 9 months. Pickering et al. conducted a meta-analysis and reported that the 3-month fall rate was 21% among Parkinson's disease patients without prior falls [13]. The included patients' background was similar with our study, as all patients were Hoehn–Yahr stage I or II at entry. In the present study, GP included not only falls but also the introduction of the necessity for gait assistance and the occurrence of freezing of gait. The relationship between the LED and falls has rarely been reported. For example, Almeida et al. analyzed 130 patients with a mean age of 70.3 ± 6.7 years and reported that fallers had a mean LED of 645.9 ± 289 mg at baseline, which was higher than non-fallers [14]. Furthermore, Hiorth et al. analyzed 211 patients with a mean age of 70.4 ± 8.3 years and found that a higher LED was associated with falls [15]. In addition, Okuma et al. reported that patients with Parkinson's disease fell mostly during the ON period [16]. Finally, Wilson et al. analyzed 109 Parkinson's disease patients with a mean age of 67.4 ± 9.9 years and a mean LED of 175 ± 132 mg and observed gait impairment over 6 years and showed that it continues to progress over this period, reflecting a combination of, and potential interaction between, disease-specific progression and age-related changes. They also found that gait impairment progresses irrespective of dopaminergic medication change [5]. Our patients in Group 3 experienced GP early, partly due to age-related changes and partly due to symptoms that were resistant to dopaminergic medication change. Regarding freezing of gait, as Virmani et al. [17] reported that it appears at 9.3 years on average from the initial motor symptoms, it is not an early symptom, which is concordant with our observation that only a minority of patients experienced freezing of gait in this study.

A further possible explanation for the relation between dopaminergic medication and GP may be the dopamine overdose hypothesis [6, 18]. Dopaminergic medication can alleviate the motor symptoms in Parkinson's disease; however, it may worsen cognitive function [6, 7, 18, 19]. Mirabella et al. showed that dopaminergic therapy selectively impairs reactive inhibition in patients with Hoehn–Yahr stage I and proactive inhibition in patients with Hoehn–Yahr stage II. Dopaminergic therapy overdoses the relatively preserved ventral striatum and its prefrontal projections, therefore impairing cognitive function [19]. Regarding freezing of gait, Cohen et al. showed that it is associated with a specific inability to appropriately engage and release inhibition [7]. Martini et al. reported that patients with mild Parkinson's disease have worse cortical sensorimotor inhibition, cognition, gait, and sway than older adults. Impaired inhibition related to increased gait variability and postural sway causes falls in patients with Parkinson's disease [8].

Another important finding from this study is that the LED24m was significantly higher in Group 2, who experienced MC first, than in Group 1, who did not experience GP or MC. Although MC included ON/OFF fluctuations and dyskinesias in this study, only one of the 40 patients in Group 2 experienced dyskinesias earlier than ON/OFF fluctuations. Therefore, almost all patients in Group 2 experienced ON/OFF fluctuations first. The pathophysiology of ON/OFF fluctuations in the striatum has not been well documented. The most accepted theory is that dopamine terminals newly formed by sprouting have altered functions, that is, decreased dopamine uptake transporter function, and that they contribute to the dysregulation of striatal dopamine release that is responsible for the emergence of wearing off [20]. The speed at which this presynaptic change occurs may vary from patient to patient. We speculate that disease progression will be quicker in patients whose presynaptic changes deteriorate faster. However, we did not score disease severity, for example, by the Movement Disorder Society-sponsored revision of the Unified Parkinson's Disease Rating Scale part III [9], so we could not measure the speed of disease progression in each patient. It is undetermined whether the patients experienced MC as we increased the LED or if we increased the LED as we observed the patients experiencing MC.

**Figure 3:**
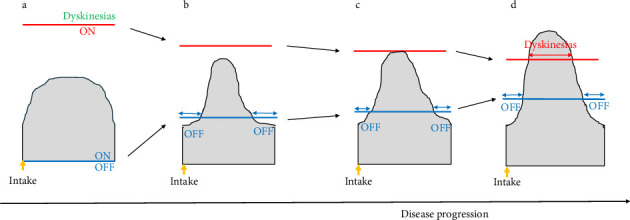
Schematic representation of a levodopa response over time. (a) No gait problems or motor complications. (b) OFF periods. (c) Shorter OFF periods. (d) Dyskinesias. Red and blue bars indicate the threshold for dyskinesias and OFF periods, respectively.

At the introduction of medical treatment and when the patients did not experience GP or MC, the medication concentration curve was shaped like an inverse U, placing it between the threshold of dyskinesias and the threshold of the OFF periods, which constitutes the therapeutic window (Figure 3(a)) [21]. As the disease progressed, the therapeutic window narrowed; in other words, not only did the threshold for dyskinesias moved downward but the threshold for the OFF periods also moved upward. The curve then changed to a bell shape. The threshold for the OFF periods moved upward and surpassed the medication concentration, resulting in OFF periods occurring at the beginning and end doses. Conversely, the threshold for dyskinesias moved downward; however, the top of the medication concentration did not reach the threshold for dyskinesias, and no dyskinesias occurred (Figure 3(b)). When the LED was increased, the patients experienced shorter OFF periods but no dyskinesias. The threshold for the OFF periods moved upward, but the medication concentration levels also increased; therefore, the patients experienced shorter OFF periods. The threshold for dyskinesias moved downward; however, the top of the medication concentration did not surpass the threshold for dyskinesias, and no dyskinesias occurred (Figure 3(c)). As the disease progressed further, the curve was still bell shaped, but taller, and the threshold for dyskinesias moved downward further; therefore, the medication concentration passed the threshold for dyskinesias. At this point, the patients experienced dyskinesias (Figure 3(d)). This theory explains why patients experienced OFF periods earlier than dyskinesias. Among the patients in Group 1, the shape of the medication concentration curve may be similar to that shown in Figure 3(a). The threshold for the OFF periods moved upward during the disease course, and it should be higher than an LED of 300 mg.

Poewe et al. reported that fluctuations and dyskinesias are problems in the mid stage of Parkinson's disease, while gait disorders and falls are problems in the late stage [22]. However, our results showed that they started to occur much earlier than the mid or late stage, and they may be observed in the early stage. Considering medical treatment without disabling the motor symptoms or MC, there may be an optimal treatment for early Parkinson's disease. Although our results showed that there were medication doses at which the patients did not experience GP or MC for 24 months, there may be a treatment that not only delays patients experiencing GP or MC for as long as possible but also that keeps patients as mobile as possible without causing MC.

One of the limitations of this study was that the patients were relatively old, and the results may not be applicable to younger patients with Parkinson's disease. In addition, we did not assess disease severity; therefore, we did not examine the speed of disease progression.

## 5. Conclusions

We observed patients who experienced neither GP nor MC for 24 months after the introduction of medical treatment. The median LED at 24 months in Group 1 was 250 mg, and this LED was between the median LED of Groups 2 and 3, namely, 300 and 225 mg, respectively. The optimal medical treatment that should be sought may be one that not only delays patients from experiencing GP or MC for as long as possible but also keeps patients as mobile as possible without experiencing MC.

## Figures and Tables

**Table 1:** Participant characteristics in Groups 1–3.

Group	1 (*n* = 25)	2 (*n* = 40)	3 (*n* = 54)	*p*
Male/female	10/15	18/22	32/22	0.198
Age at onset (years)^#^	69 (63–77)	75 (71–77)	69 (62–75)^$^	< 0.05
Hoehn and Yahr stage I/II	11/14	11/29	25/29	0.782
Orthopedic comorbidity	4	7	7	0.782
Time from onset to treatment introduction (months)	10 (3–24)	10 (3–20)	12 (3–19)	0.728
Time from onset to diagnosis (months)	5 (2–12)	8 (3–17)	8 (3–18)	0.369
LEDi (mg)	150 (100–150)	100 (100–150)	100 (100–150)	0.512
LDi (mg)	150 (100–150)	150 (100–150)	100 (100–150)	0.904
Levodopa intake/day	3 (2–3)	3 (2–3)	2 (2–3)	0.471
Started treatment with levodopa formula (*n*)	24	38	49	0.595

*Note:* Data are number of patients, numbers, or median (interquartile range).

^#^Significant difference between Groups 1–3.

^$^Significant difference with Group 2. LDi, levodopa dose at the introduction of medical treatment; LEDi, levodopa equivalent dose at the introduction of medical treatment.

**Table 2:** Levodopa intake/day during treatment course in Groups 1–3.

Group	1 (*n* = 25)	2 (*n* = 40)	3 (*n* = 54)	*p*
At stb	3 (2–3)	3 (3–3)	3 (2–3)	0.992
At 6 months	3 (3–3)	3 (3–3)	3 (3–3)	0.710
At 12 months	3 (3–3)	3 (3–3)	3 (3–3)	0.586
At 18 months	3 (3–3)	3 (3–4)	3 (3–3)	0.692
At 24 months	3 (3–3)	3 (3–4)	3 (3–3)	0.923

*Note:* Data are median (interquartile range). stb, the point at which the dosage was stabilized.

## Data Availability

The data that support the findings of this study are available from the corresponding author upon reasonable request.

## Supporting Information

Supporting information include: Supporting Figure 1: Clinical course and endpoint, definition of the LED at the endpoint, and summary of the categorization of nine representative patients into Groups 1–3; Supporting Table 1: Levodopa equivalent dose during treatment course in Groups 1–3; Supporting Table 2: Levodopa dose during treatment course in Groups 1–3; and STROBE checklist.
