# Supplementary material for: Occurrence of Motor Complications and Gait Problems After Introduction of Medical Treatment in Parkinson's Disease
Source: Parkinsons Dis. 2025 Nov 7;2025:8857969. doi: 10.1155/padi/8857969 (PMC12618128; doi:10.1155/padi/8857969)
Supplement: Supporting Information — Additional supporting information can be found online in the Supporting Information section. [file 8857969.f1.zip › Osaki-2nd Parkinson's D SupFig1.pptx]

## Slide 1
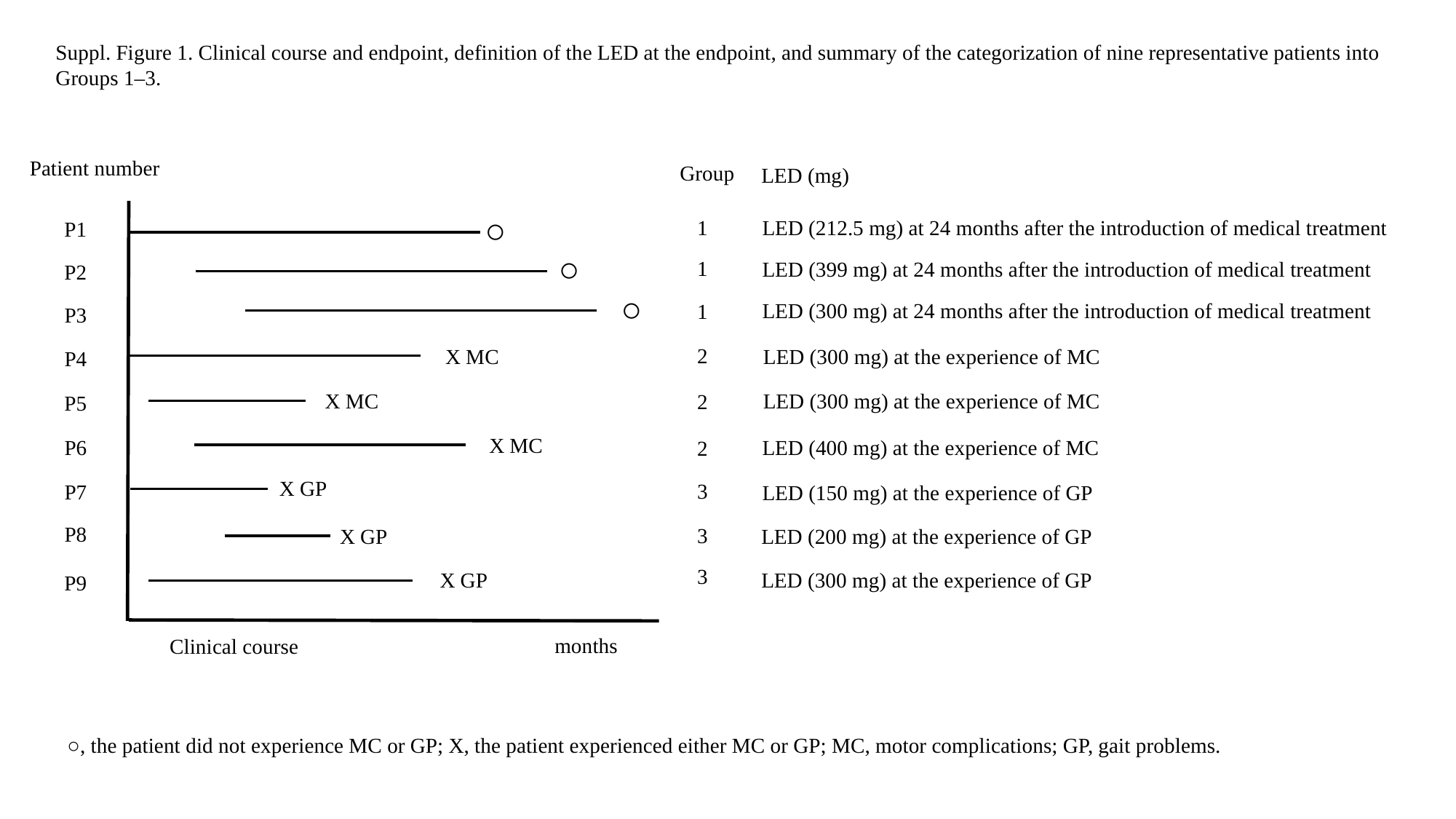

Suppl. Figure 1. Clinical course and endpoint, definition of the LED at the endpoint, and summary of the categorization of nine representative patients into Groups 1–3.
 Patient number
 Group
LED (mg)
1
LED (212.5 mg) at 24 months after the introduction of medical treatment
P1
 〇
1
LED (399 mg) at 24 months after the introduction of medical treatment
〇
P2
〇
LED (300 mg) at 24 months after the introduction of medical treatment
1
P3
2
LED (300 mg) at the experience of MC
X MC
P4
LED (300 mg) at the experience of MC
X MC
2
P5
X MC
P6
LED (400 mg) at the experience of MC
2
X GP
3
P7
LED (150 mg) at the experience of GP
P8
3
LED (200 mg) at the experience of GP
X GP
3
LED (300 mg) at the experience of GP
X GP
P9
months
Clinical course
○, the patient did not experience MC or GP; X, the patient experienced either MC or GP; MC, motor complications; GP, gait problems.
