# Supplementary material for: Occurrence of Motor Complications and Gait Problems After Introduction of Medical Treatment in Parkinson's Disease
Source: Parkinsons Dis. 2025 Nov 7;2025:8857969. doi: 10.1155/padi/8857969 (PMC12618128; doi:10.1155/padi/8857969)
Supplement: Supporting Information — Additional supporting information can be found online in the Supporting Information section. [file 8857969.f1.zip › Osaki-2nd-Parkinson's D SupTabs.docx]

Suppl. Table 1. Levodopa equivalent dose during treatment course in Groups 1‒3.

| Groups | 1 (*n* = 25) | 2 (*n* = 40) | 3 (*n* = 54) | *p* |
| --- | --- | --- | --- | --- |
| LEDstb (mg) | 150 (100‒250) | 250 (150‒300) | 150 (150‒250) | 0.142 |
| LED6m (mg) | 200 (150‒300) | 299 (200‒300) | 200 (150‒291.75) | 0.138 |
| LED12m (mg) | 200 (150‒300) | 300 (200‒400) | 200 (150‒300) | 0.142 |
| LED18m (mg) | 250 (168.25‒300) | 300 (250‒350) | 240 (150‒300) | 0.067 |
| LED24m (mg) | 250 (200‒399) | 300 (200‒350)^$^ | 225 (150‒300) | <0.05 |

Suppl. Table 2. Levodopa dose during treatment course in Groups 1‒3.

| Groups | 1 (*n* = 25) | 2 (*n* = 40) | 3 (*n* = 54) | *p* |
| --- | --- | --- | --- | --- |
| LEDstb (mg) | 150 (100‒250) | 200 (150‒300) | 150 (150‒250) | 0.113 |
| LED6m (mg) | 200 (112.5‒250) | 200 (150‒300) | 200 (150‒262.5) | 0.092 |
| LED12m (mg) | 200 (150‒300) | 250 (200‒300) | 200 (150‒262.5) | 0.141 |
| LED18m (mg) | 200 (150‒250) | 250 (200‒300) | 200 (150‒250) | 0.071 |
| LED24m (mg) | 250 (150‒300) | 250 (200‒300) | 200 (150‒250) | 0.061 |
